# A Review of the Developmental Processes and Selective Pressures Shaping Aperture Pattern in Angiosperms

**DOI:** 10.3390/plants11030357

**Published:** 2022-01-28

**Authors:** Beatrice Albert, Alexis Matamoro-Vidal, Charlotte Prieu, Sophie Nadot, Irène Till-Bottraud, Adrienne Ressayre, Pierre-Henri Gouyon

**Affiliations:** 1 CNRS, AgroParisTech, Ecologie Systématique et Evolution, Université Paris-Saclay, 91405 Orsay, France; alexis.matamoro-vidal@pasteur.fr (A.M.-V.); charlotte.prieu@uvsq.fr (C.P.); sophie.nadot@u-psud.fr (S.N.); 2 Muséum National d’Histoire Naturelle, CNRS, Institut de Systématique, Évolution, Biodiversité, Sorbonne Université, 75006 Paris, France; pierre-henri.gouyon@mnhn.fr; 3 CNRS, GEOLAB, Université Clermont Auvergne, 91405 Clermont-Ferrand, France; irene.till@uca.fr; 4 INRAE, CNRS, AgroParisTech, Génétique Quantitative et Evolution-Le Moulon, Université Paris-Saclay, 91190 Gif-sur-Yvette, France; adrienne.ressayre@universite-paris-saclay.fr

**Keywords:** cytokinesis, microsporogenesis, pollen, aperture pattern, evo-devo

## Abstract

Pollen grains of flowering plants display a fascinating diversity of forms. The observed diversity is determined by the developmental mechanisms involved in the establishment of pollen morphological features. Pollen grains are generally surrounded by an extremely resistant wall displaying apertures that play a key role in reproduction, being the places at which pollen tube growth is initiated. Aperture number, structure, and position (collectively termed ‘aperture pattern’) are determined during microsporogenesis, which is the earliest step of pollen ontogeny. Here, we review current knowledge about aperture pattern developmental mechanisms and adaptive significance with respect to plant reproduction and how advances in these fields shed light on our understanding of aperture pattern evolution in angiosperms.

## 1. Introduction

Pollen grains, the male gametophytes of flowering plants, are simple organisms composed of two to three cells surrounded by a complex multilayered protective wall made of sporopollenin (outer wall, called exine), and cellulose and pectins (inner wall, called intine). Apertures are the areas where the exine is thinner or even lacking. A great diversity in pollen grain morphology is observed in angiosperms. Variation concerns size, shape, aperture pattern, and pollen wall ornamentation [1]. Aperture pattern is defined as the number, structure, and position of apertures. Apertures may vary in structure (pore, furrow, or both), number (from no aperture to more than one hundred), and position on pollen surface. They are flexible and permeable areas preventing pollen wall breakage during volume variation due to water flows, and they also allow gas exchanges and are thereby strongly involved throughout the processes of fertilization, from pollen survival during pollination to germination of the pollen tube [2].

A wide range of variation in aperture pattern is observed throughout angiosperms [3,4]. Although variation occurs at any taxonomic level, even down to the intraindividual level [5], large categories can be recognized within angiosperms according to the predominant aperture type. Early-diverging angiosperms and monocots (as gymnosperm) mainly produce monosulcate pollen grains, i.e., a single furrow-shaped aperture located at the distal pole, while eudicots tend to produce tricolpate pollen grains, i.e., three apertures equally distributed along the equatorial region [6].

In this paper, we review the current knowledge concerning aperture pattern developmental processes and evolution. The distribution of aperture pattern diversity that may be observed today in angiosperms is the result of developmental features combined with the action of selection and constraints. The aperture pattern results from a combination of two developmental processes: one that concerns the formation of each individual aperture (structural features of the pollen wall) and the other one that determines the position and number of apertures on the pollen surface (architectural features). Selection and constraints may act on either or both developmental processes but also on the result of these developmental processes, i.e., on pollen grain morphology.

## 2. Aperture Formation

Microsporogenesis (male meiosis of seed plants) is the earliest step of pollen development. It consists of nuclear divisions associated with cytoplasmic partitioning (cytokinesis). This process starts from pollen mother cells (or microsporocytes) enclosed in a callose envelope within which meiosis takes place. Cytokinesis is achieved by the formation of callose cleavage walls. Once meiosis is completed, the resulting four microspores form a tetrad embedded within the callose wall of the pollen mother cell, until digestion of the callose by an enzyme called callase. In most species, the apertures are already visible at the late tetrad stage, showing that aperture pattern is determined during microsporogenesis.

As mentioned above, the pollen wall is complex and multilayered. The outer wall (exine) is composed of sporopollenin and is structured in several layers each with specific features. The inner wall (intine) is composed of cellulose and pectins. It is apposed to the plasma membrane of the vegetative cell. The apertures correspond generally to areas where the exine is absent or modified and the intine is thicker. Understanding how individual apertures are formed at the microspore stage thus requires understanding how the exine is formed and either inhibited or modified in the areas that will become apertures. Nevertheless, in few cases reported, the aperture develops independently in the intine and in the exine. This the case of inaperturate pollen grains which were found to have no ectoaperture but present endoaperture (Zavata et al., 1997; Pozhidaev, 2003).

Several studies have permitted to build a model of exine formation [2,7,8,9,10,11,12,13,14,15,16,17]. The model of exine formation has several steps: (1) at the tetrad stage, the microspores are entirely enclosed in callose, primexine (exine precursor) is deposited between callose and the plasma membrane; (2) the plasma membrane undulates, and structural elements (probaculae) are formed above the protrusions of the undulating membrane; and (3) sporopollenin is then deposited on the microspore surface. It has to be noted that the apertural regions are not mentioned in this model.

### 2.1. Cellular Components Correlated with Aperture Location

At the apertural sites, the absence of exine formation is generally due to an absence of primexine. This absence of primexine could, in turn, be due to the apposition of a plate of endoplasmic reticulum onto the plasma membrane, which prevents the local deposition of primexine, or it could be due to the presence of callosic knobs (also called additional callose deposits) at the places where the apertures are located. The apposition of an endoplasmic reticulum shield against the inner side of the plasma membrane in the apertural region has been observed in a large number of species. These species, which belong to both monocotyledons and eudicotyledons, exhibit various pollen aperture patterns: polyporate pollen grains—*Silene pendula* [18], monoporate pollen grains—*Zea mays* [19] and *Sorghum bicolor* [20], monosulcate pollen grains—*Lilium longiflorum* [21], tricolporate pollen grains—*Tragopogon porrifolius* [22], and tricolpate pollen grains—*Helleborus foetidus* [23] and *Helianthus annuus* [24].

**Figure 1:**
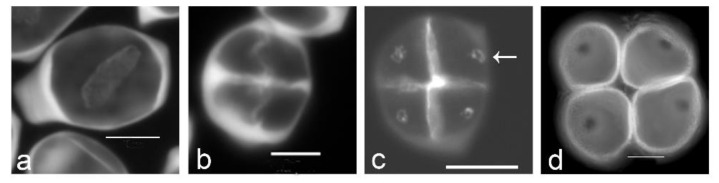
Aperture localization is associated with callose spots in *Typha latifolia*. (**a**) Successive cytokinesis with centrifugal cleavage wall formation. (**b**) Tetragonal tetrad right after cleavage wall formation. (**c**) Later-stage tetrad with callose spots resulting from additional callose deposition after cleavage wall formation (arrow). (**d**) Mature tetrad of monoporate pollen grains, callose was dissolved. Tetrads were stained with aniline blue. Scale bars: 10 µm.

The presence of callosic knobs or additional callose deposits that could prevent primexine deposition has been observed in several lineages of angiosperms, including early-diverging angiosperms. These additional callose deposits follow cytoplasm partitioning during the formation of microspores. Several callose deposits can occur successively in a species. The localization of the last additional callose deposition is always correlated with the position of the future aperture (Figure 1). Callose deposits related to aperture position were described for the first time in *Ipomoea purpurea* [25] that produces polyporate pollen grain. Later on, Blackmore and Barnes [23] showed that there is a link between differential tetrad callose wall deposition and primexine localization in *Tragopogon porrifolius*. Effectively, after the completion of the precisely structured callose wall where the positions of future ridges, spines and apertures are evident, the deposition of primexine begins. Primexine deposition is restricted to developing ridges and spines and is absent from apertural regions. More recently, the existence of a correlation between the location of the last callose deposits and the location of apertures has been demonstrated in an array of species belonging to various families in the major clades of angiosperms (magnoliids, monocots, and eudicots) and with various aperture patterns. This has been described for five eudicot species, namely *Grevillea rosmarinifolia* [26], *Paranomus reflexus* (Proteaceae), *Epilobium roseum* (Onagraceae) [27], *Protea lepicarpodendron*, and *Helleborus foetidus* (Ranunculaceae) [28], all of which produce triaperturate pollen grains. This correlation has also been observed in 28 monocot species that produce diporate, monosulcate, trichotomosulcate, tetraporate, and monoporate pollen grains [27,28,29,30,31]. These species belong to various unrelated orders and families: Butomaceae (Alismatales), Agavaceae, Amaryllidaceae, Asparagaceae and Xanthorrhoeaceae (Asparagales), Liliaceae (Liliales), Bromeliaceae and Typhaceae (Poales), and Pontederiaceae (Pontederiales). Furthermore, among early-diverging angiosperms the species *Calycanthus floridus* (Calycanthaceae, magnoliids) that produces disulculate pollen grains also exhibits this correlation [30]. Thus, the correlation between the localization of the last additional callose deposition and the position of the future aperture is not linked to a particular aperture pattern.

Additional alternative ontogenic processes correlated with aperture formation and positioning have been described at the intracellular level in a few species. In *Epilobium angustifolium*, it has been shown that interstitial bodies are present at the future apertural sites [32]. In *Parkinsonia aculeata*, the plasma membrane of each microspore presents an irregular pattern except at the apertural site where the membrane is smooth [33]. In *Liriodendron tulipifera*, *Nelumbo nucifera*, and *Nelumbo lutea*, aperture formation is not the result of primexine formation inhibition at the future apertural sites. The microspores of *Liriodendron tulipifera* are totally enclosed in exine, and the aperture position seems to be determined by an exine fold localized in the distal region of the tetrad. The exine fold breaks down at microspore liberation from the tetrad [34]. In *Nelumbo nucifera*, Flynn and Rowley [35] state that presumptive apertural areas do not exist in early microspores of this species because primexine is deposited overall the microspore surface, and they could not observe any change that could permit to detect how or when apertures form. The appearance of the colpi might be an example of focal autolysis. In *Nelumbo lutea*, Kreunen and Osborn [36] describe primexine distribution uniformly around the microspore, and no accumulation of reticulum endoplasmic at the apertural sites. They confirm the post-tetrad establishment of aperture in *Nelumbo*.

Recent studies have highlighted the role of specific proteins in the process of aperture formation and localization. In *Arabidopsis thaliana*, rice, and maize, the loss of the INP1 protein causes a complete aperture loss, suggesting a role of INP1 as an aperture factor [37,38,39]. The role of INP1 is conserved in *Eschscholzia californica* [40]. INP2 has been identified as a partner of INP1 [41], and *inp2* mutant displays inaperturate pollen grain. INP1 and INP2 are both proteins of unknown function [41]. The *Arabidopsis thaliana* protein kinase D6PKL3 is also involved in aperture formation [42]. Indeed, during pollen development, D6PKL3 accumulates at the future aperture sites. The *d6pkl3* mutants develop abnormal apertures on the pollen surface, resulting in pollen grains that either lack apertures or, more commonly, have aperture regions that are partially covered with exine [42]. A lectin receptor-like kinase in *Oryza sativa*, OsDAF1 is also essential for aperture formation [39]. This protein localizes to the future aperture sites at the tetrad stage. Most pollen grains produced by this mutant were aborted, and the surviving pollen grains were inaperturate [39]. A recent study concerning the *BcMF8* transgenic line of *Brassica campestris* [43] has revealed the involvement of the BcMF8 arabinogalactan protein in cell wall development, aperture formation, and pollen tube growth. BcMF8 is a cell-wall-secreted protein which acts to maintain normal intine formation. In the transgenic line, intine is thicker both at the apertural and in non-apertural sites and 80% of pollen grains are tetra-aperturate instead of triaperturate. More information is needed to understand the role of this protein, as microsporogenesis was not described in the paper.

### 2.2. Determination of Aperture Localization

Cellular mechanisms and the position of cellular components involved in aperture formation in the microspores were explored early in the 20th century by Wodehouse [44] followed by Blackmore and Crane [45] and Ressayre et al. [46]. They suggested that the spatial information determining aperture localization within the tetrad is provided by the last contact points persisting at the end of cytokinesis between the cytoplasms of the future microspores. Ressayre et al. [47] proposed a model that predicts these last contact point positions between microspores. This model is based on the interaction among three meiotic characters: the type of cytokinesis, the tetrad form (which results from the respective orientation of the second meiotic axes), and the way in which callose cleavage walls are formed among the microspores. Cytokinesis type (successive/simultaneous) and tetrad form (tetragonal/rhomboidal/tetrahedral) determines the number and the spatial arrangement of cleavage walls among nuclei. The mode of cleavage wall formation (centrifugal/centripetal callose progression within a cleavage plan), associated with the number and spatial distribution of cleavage walls, and with the timing of cleavage walls formation, determines the areas where the callose is deposited last within the tetrad. These areas are the last contact points among the four microspores. Ressayre et al. [47] furthermore proposed two different ways in which aperture position may be determined. In the first one, the apertures are found at these last contact points among microspores, as suggested by Wodehouse [44] (grouped apertures), while in the second one, the apertures are centered on the distal poles of the microspores and oriented toward these last points (polar apertures) [47]. This model accounts for the localization of apertures only when aperture number is comprised between one and four. The last contact points among microspores are potentially determined by microtubule distribution, which directs the transport of cellular components to the places where apertures should be formed [48].

The developmental model determining aperture localization [47] has been tested by examining the role on aperture pattern of cytokinesis, tetrad form, and callose cleavage wall formation in a large array of species with various aperture patterns. Examples are detailed in what follows.

#### 2.2.1. Role of Cytokinesis in Aperture Pattern Determination

**Figure 2:**
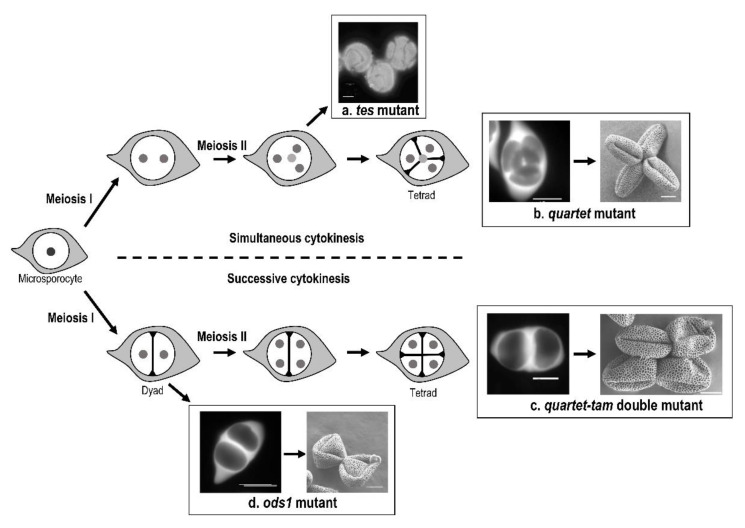
Cytokinesis type affects aperture pattern in *Arabidopsis thaliana*. (**a**) Absence of cytoplasm partitioning in the *tes* mutant, resulting in pollen grains display abnormal circular aperture pattern. (**b**) Simultaneous cytokinesis in the *quartet* mutant, with formation of tetrahedral tetrads. The resulting tricolpate pollen grains are shed in tetrads in which all colpi meet toward the center of the tetrad, with each aperture being in contact with the aperture of another pollen grain. (**c**) Successive cytokinesis in the *quartet-tam* double mutant. The tricolpate pollen grains are arranged in quadratic tetrads. The apertures of the two pollen grains separated by the second meitotic division are associated in pairs. (**d**) Successive cytokinesis in the *osd1* mutant failing after the first meiotic division. Pollens grains display 8 apertures. Nuclei are represented by small grey circles (nuclei that are behind the others are in lighter grey, and nucleus in the microsporocyte is diploid), callose walls are represented by black lines, and callose outer wall is in light grey. Scale bars: 10 µm.

The first report of the role of cytokinesis in the determination of the aperture pattern was through the analysis of the *tes*/*stud* cytokinetic mutants of *Arabidopsis thaliana* [49,50,51]. These mutants present as a primary defect a failure in cytokinesis: the four meiotic nuclei remain within the same cytoplasm (Figure 2a). Therefore, a single microspore is formed and produces a single pollen grain possessing four nuclei. These mutants display extra-apertures in abnormal orientations on the pollen wall, instead of the three colpi that characterize wild-type pollen. The absence of cytoplasm partitioning in *tes*/*stud* mutants has multiple consequences, which prevents us from identifying clearly which factors are directly involved in aperture modification. In Albert et al. [52], two mutant strains of *Arabidopsis thaliana*, *quartet*, and *quartet-tam* differing only in the type of cytokinesis during microsporogenesis, simultaneous in *quartet* and successive in *quartet-tam*, demonstrate the impact of a change from simultaneous to successive cytokinesis in aperture pattern ontogeny (Figure 2b,c). In the *quartet* mutant, pollen grains are incompletely separated at the end of microsporogenesis, and pollen grains are shed in tetrads, but the aperture number and distribution are unaltered. While the number of apertures is not affected in the *tam* mutant [53], the arrangement of the three apertures within the tetrad is modified compared to the wild type as can be seen by the comparison of the tetrad between quartet and quartet-tam tetrads. Indeed, in the quartet mutant, the three apertures of each microspore are each paired with an aperture of the three other microspores of the tetrad. By contrast in the quartet-tam mutant, the three apertures of each microspore are paired with those of its sister microspore separated by the second meiotic. While the tam mutant produces tetrads of haploid microspores, the cytokinesis mutant *osd1* [54] does not undergo the second meiotic division and produces dyads of diploid pollen grains instead of tetrads (Figure 2d). The aperture pattern of the resulting pollen grains is modified compared to the wild type since pollen grains are 4-8-aperturate [55,56].

#### 2.2.2. Role of Tetrad Form in Aperture Pattern Determination

**Figure 3:**
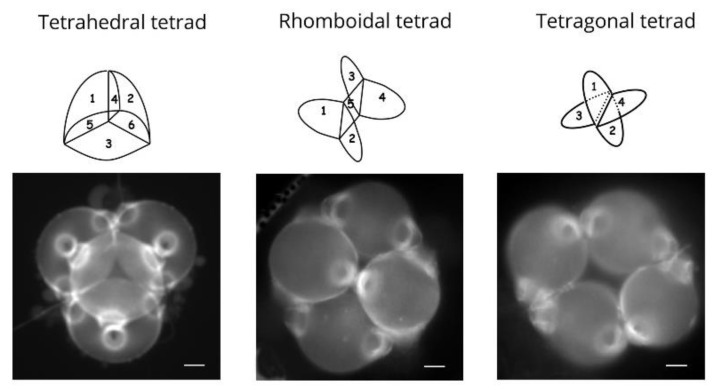
Tetrad form is associated with aperture pattern in *Epilobium roseum*. Tetrahedral, rhomboidal, and tetragonal tetrads of pollen grains. In the tetrahedral tetrads, microspores are separated by six callose cleavage walls; the pollen grains are triporate and shed in tetrads. In the rhomboidal tetrads, microspores are separated by five callose cleavage walls; the pollen tetrad is composed of two triporate pollen grains and two diporate pollen grains. In the tetragonal tetrads, microspores are separated by four callose cleavage walls; pollen grains are all diporate. Scale bars: 20 µm.

Tetrad form is determined by the localization of nuclei within the microsporocyte [46,57,58,59]. Studies have examined the effect of meiotic disturbance by drugs or centrifugation on aperture type, in wheat [57] and *Lilium henryi* [58,59] which both have pollen grains with a single distal aperture (a pore in the case of wheat and a furrow in the case of *Lilium henryi*). These studies have showed that distal polar aperture formation is dependent on the position of nuclei in the cytoplasm which in turn affects tetrad form. Variation in the shape of the tetrad also leads to changes in the number of cleavage walls separating the microspores, and therefore to the number of last contact points persisting at the end of cytokinesis. In *Nicotiana tabacum* cv. Amballema, an increase in cleavage wall number during cytokinesis, which is a modification of the tetrad form, is correlated with an increase in microspore aperture number [46]. A study in *Epilobium roseum* (Onagraceae) and *Paranomus reflexus* (Proteaceae) further demonstrated the role of tetrad form in aperture pattern ontogeny [60]. Both species produce pollen grains shed in tetrads that may present three different forms within the same anther: tetragonal, rhomboidal, and tetrahedral, with this latter type representing the majority of observed tetrads. Both *E. roseum* and *P. reflexus* display pollen heteromorphism since they simultaneously produce pollen grains with two or three apertures. Tetrahedral tetrads are composed of four triaperturate pollen grains, rhomboidal tetrads are composed of two triaperturate pollen grains and two diaperturate pollen grains, and tetragonal tetrads display four diaperturate pollen grains (Figure 3). The differences in aperture numbers are therefore directly associated with variation in tetrad form.

#### 2.2.3. The Role of Polyploidy or Hybridization in Aperture Patterns

An increase in ploidy number has been shown to increase aperture number [61,62,63,64,65]. This effect of ploidy on aperture number is certainly largely due to meiotic anomalies. The meiotic anomalies are common in both polyploids and hybrids resulting in the presence of micronuclei in the microspores in addition to the main nucleus or in abnormal microtubule furrows leading to an increase in the last contact points [46,48].

#### 2.2.4. Role of Callose Cleavage Wall Formation in Aperture Pattern Determination

**Figure 4:**
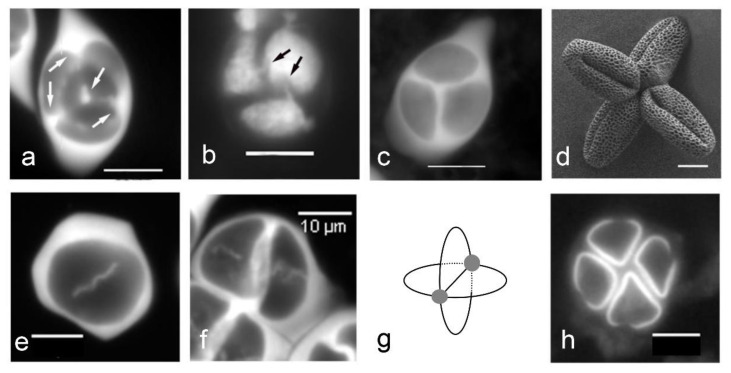
Callose cleavage wall formation determines aperture localization in *Arabidopsis thaliana* and *Asparagus officinalis*. (**a**–**d**) *Arabidopsis thaliana*. (**a**) Centripetal cleavage wall formation: callose deposition starts from the center of the tetrad and from the border of each cleavage plane (arrows). (**b**) Last contact points between microspores (arrows). (**c**) Tetrahedral tetrad. (**d**) Triaperturate pollen grains with apertures localized at the last contact points. (**e**–**h**) *Asparagus officinalis*. (**e**) Centrifugal cleavage wall formation. (**f**) Successive cytokinesis. (**g**) Schema of cleavage walls showing the last points of contact between the microspores (grey circles). (**h**) Monosulcate pollen grains, with the extremities of the sulcus oriented toward the last contact points among microspores. Scale bars: 10 µm.

The last points of contact between microspores correspond to the last points covered by callose during the cleavage wall formation. Cleavage wall formation can be centripetal or centrifugal, and the last points of callose deposition are different in number and position in these various situations. In *Arabidopsis thaliana*, the formation of the cleavage walls is centripetal, and the apertures are localized at the last contact point between microspores (Figure 4). In *Nicotiana tabacum*, where the formation of the cleavage walls is also centripetal, Ressayre et al. [48] have also observed a correlation between the number of last contact points and aperture number. In *Asparagus officinalis*, cleavage wall formation is centrifugal, and the apertures are oriented toward the last contact points (Figure 4g,h).

The involvement of the last contact points in the determination of aperture localization is possible only for low aperture numbers which correspond to the main pollen morphologies found in angiosperms. A major global argument in favor of the role of cytokinesis, cleavage wall formation, and tetrad shape, i.e., the last contact points, in aperture determination is the study of Matamoro et al. [66]. In this study, several eudicot species with inaperturate pollen and their sister species with triaperturate pollen were compared. They found conserved developmental pathway of cytokinesis type, cleavage wall formation, and tetrad shape leading to three last contact points in all triaperturate species, whereas in species with inaperturate pollen grain, these characters are very variable. The authors claim that if these developmental characters were not involved in the determination of triaperturate morphology, they should vary as in inaperturate species.

## 3. Selection and Developmental Constraints Involved in the Evolution of Pollen Aperture Pattern

Plant male success depends on the survival and the efficiency of fertilization by the male gametes carried by pollen grains, which in turn depends on numerous factors [67]. Some of these factors such as germination capacity and survival, as well as the harmomegathic properties of the pollen wall (the ability to absorb bending stresses occurring during desiccation and hydration [44]) may depend on pollen grain aperture number. The fossil record shows that the first angiosperm pollen morphs had only one aperture and that pluriaperturate pollen grains are derived morphs [6,68,69]. Furthermore, an increase through evolutionary time in the speed of pollen tube growth has been reported [70,71] together with an increase in life expectancy of pollen grains [70,72]. All these studies suggest that selection has likely played a role in the evolution of aperture pattern.

The possible roles of selection and/or developmental constraints in the evolution of aperture pattern were studied at the scale of angiosperms as a whole. In angiosperms, even if variation in aperture pattern may be observed, two stases (character states that represent an evolutionary equilibrium) of pollen aperture pattern can be considered: the monosulcate stase characterizes basal angiosperms and monocots, while the tricolpate stase characterizes eudicots (also named tricolpate angiosperms). These two stases could be explained by selection on the aperture pattern (phenotype) or by constraints being exerted during the developmental process involved in aperture formation. Looking at the developmental stability/variation permits one to distinguish between selection and constraints. For example, in a situation where a single type of aperture pattern is observed in a clade, the occurrence of variation during microsporogenesis indicates that aperture pattern (the phenotype) is under selection in this clade. Indeed, variation in microsporogenesis offers a potential for the production of various aperture patterns, as previously mentioned. If only one phenotype is observed, this suggests that selection on aperture pattern eliminated the developmental pathways that would result in the production of other aperture patterns. By contrast, if microsporogenesis is highly stable in a given clade, this suggests that either there is selection on both aperture pattern and the developmental pathway leading to this single phenotype (the best developmental sequence to produce an aperture pattern is retained) or by the presence of developmental constraints, i.e., a single developmental pathway may lead to an aperture pattern.

The monosulcate aperture type observed in basal angiosperms and monocots is produced by different combinations of the characters involved in the determination of aperture pattern, i.e., cytokinesis type, cleavage wall formation, tetrad form, and additional callose deposition [31,73,74,75,76]. Such variation in the developmental sequence strongly suggests that this monosulcate aperture type is selected in basal angiosperms and monocots, with relaxed developmental constraints on the development. Indeed, it would theoretically be possible to produce other pollen types, but developmental sequences combining features that lead to monosulcate pollen grains are mostly retained.

**Figure 5:**
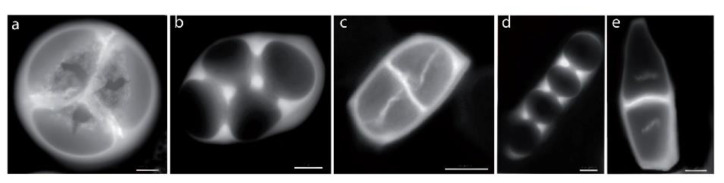
Microsporogenesis of triaperturate and inaperturate pollen grains in *Lavatera maritima*, *Reinwardtia cicanoba*, *Apocynum cannabinum*, and *Trachelospermum jasminoïdes*. (**a**) *Lavatera maritima*, microsporogenesis of triaperturate pollen grains: simultaneous cytokinesis, centripetal cleavage wall formation, and tetrahedral tetrad. (**b**) *Reinwardtia cicanoba*, (**c**) *Apocynum cannabinum*, (**d**) *Reinwardtia cicanoba*, and (**e**) *Trachelospermum jasminoïdes*. (**b**–**e**) Microsporogenesis of inaperturate pollen grains: simultaneous cytokinesis (**b**,**d**) or successive cytokinesis (**c**,**e**), centripetal cleavage wall formation (**b**,**d**) or centrifugal cleavage wall formation (**c**,**e**), and rhomboidal (**b**), tetragonal (**c**), and linear (**d**–**e**) tetrad. Scale bars: 10 µm (**a**,**b**,**d**), 5 µm (**c**,**e**).

The tricolpate pollen grains of eudicots, widely dominant in this large clade, are produced by a remarkably conserved developmental sequence: simultaneous cytokinesis, centripetal cleavage wall formation, and tetrahedral tetrad form (Figure 5a) [66,73], suggesting strong selection and/or constraints exerted during development. By contrast, in their sister species presenting pollen grains devoid of apertures (inaperturate), variation in cytokinesis, formation of cleavage wall, and tetrad form may be observed, without affecting the final phenotype. Variation in microsporogenesis in these sister species indicates the absence of developmental constraint, whereas stability in microsporogenesis reflects the presence of developmental constraints.

The first study in *Codiaeum variegatum* var. *pictum* (eudicots: Euphorbiaceae), which produces inaperturate pollen grains, revealed variation in cytokinesis type, cell wall formation, and tetrad form [77]. This study was extended to other members of the Euphorbiaceae family and afterward to the eudicots, revealing a similar level of variations in microsporogenesis (Figure 5b–e) [66,78] and showing that various developmental sequences can be achieved in this clade and therefore an absence of developmental constraints on developmental sequence. The stase in triaperturate pollen grains in eudicots is therefore mostly explained by selection on the aperture pattern.

Only a few studies have actually demonstrated that pollen aperture pattern is potentially under selection. Dajoz et al. [79,80] performed the first studies showing differential selection over pollen grain presenting different aperture pattern. This study was conducted using *Viola diversifolia,* which possesses tri- and four-aperturate pollen grains within the same anther. In this species, triaperturate pollen grains survive better but germinate slower than four-aperturate pollen grains. Later, Till-Bottraud et al. [81] have shown in *Viola calcarata* that the proportion of 4/5-aperturate pollen grains produce by plants was correlated with pollination efficiency, with greater proportions of 5-aperturate pollen when pollination was more efficient (i.e., favoring the short-lived, fast-germinating morph). In *Nicotiana tabacum*, the proportion of the different pollen morphs within plants is under genetic control [82] and thus can be modified by selection. Other studies have tested the harmomegathic properties of pollen grains differing by their aperture number. A theoretical study on pollen wall folding due to dehydration modelled monosulcate, tricolpate, inaperturate, and porate pollen grains [83]. The authors revealed the importance of aperture pattern in pollen folding, with the presence of elongated apertures being critical for achieving a predictable and reversible folding. Matamoro-Vidal et al. [84] found significant differences due to aperture pattern in the percentage of pollen with disrupted plasma membrane in osmotic stress conditions. Prieu et al. [55] studied the impact of aperture number on pollen wall resistance to osmotic stress using *Arabidopsis thaliana* mutants (*inp1*, *lsq6*, and *osd1*). They showed that an increase in aperture number is associated with an increase in pollen wall breakage. Therefore, an increase in aperture number decreases survival of pollen grains submitted to osmotic stress. Pollen grains devoid of apertures (*inap1-1*) are the best in osmotic resistance, and triaperturate pollen grains (wild type) performed significantly better than 4- (*lsq6*) and 4-8- (*osd1*) aperturate pollen grains. Using the same mutants, Albert et al. [85] suggested that triaperturate pollen grains might provide the best trade-off among various pollen performance traits, ensuring strong germination ability, high longevity, and a good-enough capacity to accommodate volume changes, thus potentially explaining the prevalence of this morphological trait in the eudicot clade.

## 4. Conclusions

The aperture pattern is a complex character. The localization of several proteins and cellular components has been shown to correlate with the future apertural site. The cytokinesis type, the tetrad form, and the callose wall formation have been shown to be implicated in the determination of the number and localization of the apertural site(s). The combination of mutant studies and comparative analyses of early pollen development support the idea that the two evolutionary stases, namely the monoaperturate and triaperturate aperture patterns, are maintained by selection during evolution.

## Data Availability

Not applicable.
